# Improved organs-of-interest dose reduction potential with intensity modulated proton therapy and breath-hold in mediastinal lymphoma

**DOI:** 10.1016/j.phro.2026.100996

**Published:** 2026-05-10

**Authors:** Bastiaan D.P. Ta, Richard A.M. Canters, Kim van der Klugt, Gloria Vilches-Freixas, Esther Kneepkens, Fleur Vereijken, Maud Cobben, Maud van den Bosch, Indra Lubken, Cissy Stultiens, Marije Velders, Tina Verstappen, Gyanne Tholen, Anne G.H. Niezink, Dirk K.M. De Ruysscher, Maaike Berbée

**Affiliations:** a Maastro Clinic, GROW Research Institute for Oncology & Reproduction, Maastricht University Medical Centre+, Department of Radiation Oncology (MAASTRO), Maastricht, the Netherlands; b University Medical Center Groningen, Department of Radiation Oncology, Groningen, the Netherlands

**Keywords:** Hodgkin disease, Lymphoma, Mediastinal neoplasms, Breath holding, Proton therapy, Organs of interest

## Abstract

**Background and Purpose:**

For mediastinal lymphoma patients, comparative data on intensity modulated proton therapy in breath-hold (IMPT-BH) versus other radiation techniques remain limited, particularly regarding dose-volume histogram (DVH) metrics and normal tissue complication probability (NTCP) for late side effects.

**Materials and Methods:**

This retrospective in silico planning study included 58 lymphoma patients, treated between 2019 and May 2024, having 4D-CT and BH-CT available. Four plans per patient were made: volumetric arc therapy in free-breathing (VMAT-FB), VMAT-BH, IMPT-FB, and IMPT-BH. An absolute dose difference ≥ 1 Gy was considered clinically relevant, and patients were assigned to the optimal technique.

**Results:**

IMPT-BH resulted in the lowest average mean heart dose (MHD) at 5.4 Gy (*p* < 0.01) compared to VMAT-FB (8.0 Gy), VMAT-BH (6.3 Gy) and IMPT-FB (5.8 Gy). IMPT-BH resulted in the lowest average mean lung dose (MLD) at 6.7 Gy (*p*<0.01), compared to VMAT-FB (9.2 Gy), VMAT-BH (7.5 Gy), and IMPT-FB (7.3 Gy). Average bilateral mean breast dose (MBD) was lower with IMPT (2.1 Gy) compared to VMAT (3.2 Gy; *p*<0.01). IMPT was optimal for ≥ 69% of patients.

**Conclusions:**

IMPT-BH achieved the lowest MHD, MLD, and bilateral MBD compared to IMPT-FB, VMAT-BH, and VMAT-FB in the majority of patients.

## Highlights

• Proton and breath-hold techniques were compared to photon and free-breathing.
• Proton breath-hold reduced mean heart and lung dose versus other techniques (*p*<0.01).
• It reduced mean heart (8.0 to 5.4 Gy) and lung (9.2 to 6.7) vs photon free-breathing.
• Proton therapy reduced bilateral mean breast dose versus photon therapy (*p*<0.01).
• Proton therapy: overall dose-related optimal technique for at least 69% of patients.

## Graphical abstract

**Figure:**
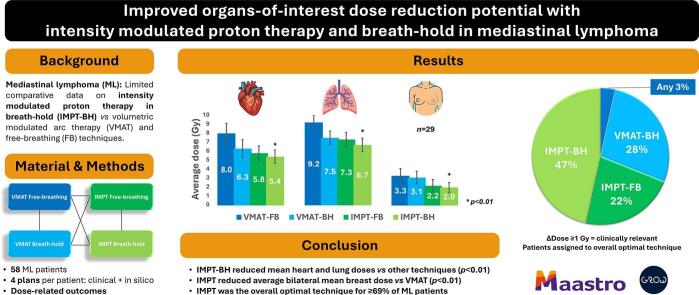

## 1 Introduction

Radiotherapy is a key curative treatment for mediastinal lymphomas (ML), which includes Hodgkin lymphoma (HL), diffuse large B-cell lymphoma (DLBCL) and primary mediastinal B-cell lymphoma (PMBCL). Early-stage ML is treated with (immuno)chemotherapy followed by radiotherapy (combined treatment modality), consolidation RT or salvage RT, resulting in favourable five-year progression-free rates ranging from 90 to 99%, and overall survival from 80 to 99% [1], [2], [3], [4], [5], [6], [7], [8], [9], [10]. Advanced stage ML patients may undergo consolidation RT following a partial metabolic response after full-course systemic therapy or salvage radiotherapy in relapsed/refractory setting [5], [9], [11], [12]. These patient groups have lower overall survival, ranging from 22 to 90%, depending on lymphoma type, degree of response, and next-line treatment options [10], [13], [14], [15]. Due to the young age (median 28 years; 21–36 IQR) of HL and PMBCL patients [16] and high overall survival rates, ML survivors face long-term treatment-related adverse events that affect quality of life and, after 20–25 years following treatment, life expectancy [16], [17], [18], [19], [20]. Important sequelae include acute coronary events (ACE) [21], cardiac valve disease [22], congestive heart failure [23], and secondary malignant neoplasms [24], [25], e.g. breast [26], [27], lung and skin cancer [28].

Therefore, the primary focus has been on minimizing treatment-related sequelae without compromising complete remission rates. Modern radiotherapy techniques have successfully reduced dose to organs-of-interest (OOIs) by improving conformality [29], [30], [31], [32]. Techniques such as deep-inspiration breath-hold (BH) and intensity-modulated proton therapy (IMPT) are increasingly becoming the standard of care.

BH leads to an anatomical shift, often increasing the distance between OOIs and the target volume [31], [33], [34]. BH also mitigates intrafraction and decreases interfraction motion, enabling a smaller PTV margin that further reduces dose to OOIs [35]. Volumetric-arc therapy with BH (VMAT-BH) reduced the mean heart dose (MHD), mean lung dose (MLD), and bilateral mean breast dose (MBD) with 2.0 Gy, 2.6 Gy and 0.6 Gy compared to VMAT in free-breathing (VMAT-FB), respectively [35]. These results are comparable to reported data in literature: photon-BH decreased the MHD with 1–3 Gy, MLD with 1–3 Gy, and MBD with 0–2 Gy, compared to photon-FB [29], [30], [32], [34], [35], [36], [37], [38], [39].

IMPT with pencil-beam scanning is an advanced radiotherapy technique used for ML patients. Compared to photon therapy, it generally delivers lower entry, exit and scattered radiation doses, reducing dose to surrounding OOIs. Single-center studies reported dose differences between proton and photon techniques, with or without BH [37], [40], [41], [42], [43], [44], [45]. No single study compared all four techniques simultaneously.

Data on dose differences in OOIs, comparing IMPT-BH with other techniques is limited and largely based on in silico plan comparison studies from centers that did not clinically implement IMPT-BH at the time of publication. Furthermore, centers that did implement IMPT-BH have not reported plan comparison results between proton and photon techniques.

With the implementation of IMPT-BH, ML patients can be treated with one of the four clinically available techniques: VMAT-FB, VMAT-BH, IMPT-FB, and IMPT-BH. This retrospective single-center in silico plan comparison study primarily aimed to report DVH-metric differences in heart, lungs, and breasts between these four techniques based on planning-CTs of ML patients. Secondary objectives were to report the normal tissue complication probability (NTCP) differences for late side effects between the four techniques, i.e. ACE risk based on the MHD, SLC on MLD, and SBC on MBD; and to analyze which technique was optimal for each patient in terms of dose.

## 2 Materials and methods

### 2.1 Patient inclusion

This retrospective single-center radiotherapy plan comparison study was approved by the institutional review board (Study Protocol 1204). Sixty-seven consecutive patients, treated with curative intent for an ML in the period of December 2019 through May 2024, were identified. Two patients were excluded due to technical treatment planning system (TPS) issues, rendering their CT scans unusable for analysis. One patient did not produce a usable 4D-CT and underwent a regular FB-CT. Six patients did not receive a BH-CT for various reasons: initial policy not requiring a BH scan for externally referred patients following IMPT-FB qualification (*n* = 3); prechemotherapy plan comparison not showing a benefit with IMPT-BH (*n* = 1); or visually estimated lack of dose benefit based on the target volume, i.e. unilateral lung hilum and para-aortal node (*n* = 2).

In conclusion, 58 of the 67 patients had both 4D-CT and BH-CTs available and were included in the study.

### 2.2 Patient and treatment characteristics

The majority of patients were treated for Hodgkin lymphoma (81%), were early-stage at diagnosis (95%), and all had mediastinal involvement (Table 1, Table 2). Early-stage patients with combined modality treatment or primary RT received 20 or 30 Gy. Patients with consolidation or salvage treatment received 36 or 40 Gy in 2 Gy per fraction equivalent dose (EQD_2_) on metabolic residual disease or progressive locations, and 30 Gy EQD_2_, electively.

**Table 1:** Patient, disease and treatment characteristics.

***Characteristic***	***N = 58***
**Sex**	
Female	29
Male	29
**Median age at start of the RT treatment**	**34 years (range 17–72)**
Hodgkin lymphoma	27 (17–72)
NLPHL	66
Primary Mediastinal B-cell lymphoma	30 (27–55)
Diffuse Large B-cell lymphoma	57 (36–66)
**Cardiovascular risk factor present**[48], [63]	
0	30 (52%)
≥1	28 (48%)
**Smoker**	
Never smoked or cessation ≥ 1 prior to RT treatment	43 (74%)
Active smoker, cessation < 1 year, ≥20 pack years	15 (26%)
**Prior RT in thoracic region**	**0**
**Diagnosis**	
Hodgkin lymphoma	47 (81%)
NLPHL	1 (2%)
Primary mediastinal B-cell lymphoma	7 (12%)
Diffuse large B-cell lymphoma	3 (5%)
**Ann Arbor stage at diagnosis**	
I	6 (10%)
II	49 (85%)
III	1 (2%)
IV	2 (3%)
**Bulky disease**	
No	30 (52%)
Yes	28 (48%)
**Treatment setting**	
Combined modality treatment	48 (83%)
Consolidation RT	7 (12%)
Salvage RT	2 (3%)
Primary RT	1 (2%)
**Prescribed RT dose**	
20 or 30 Gy (EQD_2_)	49 (84%)
36 or 40 Gy (EQD_2_)	9 (16%)
**Simultaneous integrated boost**	
No	8 (14%)
Yes	50 (86%)
**Completed radiotherapy treatment**	58 (100%)

NLPHL = Nodular lymphocyte-predominant Hodgkin lymphoma. EQD_2_ = 2 Gy per fraction equivalent dose.

**Table 2:** Anatomic regions of nodal involvement.

***Region***	***N***	***Location, n (%)***			
**Axilla**	58	**Not involved**	**Left**	**Right**	**Bilateral**
		36 (62)	8 (14)	7 (12)	7 (12)
**Lung hilum**	58	**Not involved**	**Left**	**Right**	**Bilateral**
		17 (29)	5 (9)	16 (28)	20 (34)
**Mediastinum**	58	**Not involved**	**Cranial of carina**	**Caudal of carina**	**Cranially and caudally**
		0	1 (2)	1 (2)	56 (96)
**Directly on diaphragm**	58	**Not involved**	**Involved**	**N/A**	**N/A**
		47 (81)	11 (19)		

### 2.3 Treatment preparation

Target volume delineation was performed by radiation oncologists and a physician assistant, experienced in the field of hematological malignancies. Mean clinical target volume (CTV) volumes on the 50ex phase of the 4D-CT and BH-CT were 303 ± 185 cm^3^ and 293 ± 198 cm^3^, respectively. Four treatment plans were made for each patient: VMAT-FB, VMAT-BH, IMPT-FB and IMPT-BH. Available clinical plans were used and missing plans were created in silico according to the requirements for clinical plans; all by radiation technologists (RTTs) experienced in making RT plans for ML patients.

For the VMAT plans, the (CTV) was expanded using an isotropic margin of 8 mm for FB, and 5 mm for BH, to create a planning target volume (PTV) in the intrathoracic region, with a 5 mm margin in the neck, periclavicular and axillary regions. Full-arc VMAT plans were made with the Eclipse TPS (Varian Medical Systems v.16, US). The median number of arcs was 3 (2–3) for both techniques, and all plans had one isocenter. Plans were optimized to reduce OOI doses, while ensuring ≥ 95% of prescribed dose covered ≥ 99% of the PTV.

IMPT plans with pencil beam scanning technology were made with the RayStation TPS (RaySearch Laboratories v.12A, Sweden) on the CTV, with an 8 mm robustness margin in the intrathoracic region and 5 mm for neck, periclavicular and axillary regions; 3% range uncertainty; and spot spacing of 0.3 or 0.4 times the sigma of the spot at that specific energy for each energy layer. The relative biological effectiveness (RBE) weighted dose was reported using an RBE of 1.1 for protons [46]. Reported TPS doses are biologically weighted doses, expressed in Gy (RBE). Robust optimization of the coverage in 3D was performed on the average-CT for every separate CTV subunit, i.e. neck (left and right), axilla (left and right), mediastinum (cranial and caudal of the carina) and nodes directly located on the diaphragm. For clinical plans, baseline shift evaluation was initially performed, and IMPT-FB plans were evaluated in 4D to account for potential variations caused by respiratory motion. The median number of beams was 6 (range 3–11) for IMPT-FB and 6 (3–13) for IMPT-BH. The median number of isocenters was 2 (1–4) for IMPT-FB and 2 (1–4) for IMPT-BH. These were dependent on the target volume. Plans were optimized to reduce dose to OOIs while ensuring ≥ 94% of the prescribed dose covering ≥ 98% of the CTV in the voxel-wise minimum [47]. Volumes > 107% of the prescribed dose were minimized in the nominal plan, with a guidance of 110% in the voxel-wise max plan. Dose-volume histogram (DVH) metrics of serial OOIs were evaluated on the voxel-wise max plan, and parallel OOIs on the nominal plan.

All clinical plans were approved by radiation oncologists and medical physicists experienced in photon or proton therapy for ML patients, after evaluating clinical goals. All in silico plans also met clinical goals. A detailed description of the treatment preparation can be found in Supplementary Material A-D.

### 2.4 Data collection

Patient, disease and treatment characteristics were extracted from electronic medical records. CTV volumes, integral dose (expressed in Gy/L), MHD, MLD, and MBD (for all female patients) were collected from the TPS. Clinical data and doses were used to calculate NTCP values for ACE, SLC and SBC, with models used for the Dutch model-based selection method [48], [49]. Details on the NTCP models are provided in Supplementary Material E.

### 2.5 Statistical analyses

SPSS Statistics (IBM v29, US) was used for statistical analyses. Descriptive analyses were performed for baseline characteristics. Skewness was calculated for dose-related endpoints and NTCP values. To test for global technique effect, repeated measures ANOVA or Friedman test was performed for parametric and non-parametric data, respectively. Subsequently, paired samples T-tests were used for parametric data and Wilcoxon signed-rank tests for non-parametric data to assess differences between the four RT techniques. A two-sided *p* value of < 0.05 was considered statistically significant. The Holm–Bonferroni method was applied to control the family-wise error rate across multiple hypotheses.

To analyze which technique was optimal for each patient with regard to dose, an absolute dose difference of ≥ 1 Gy between any technique was considered clinically relevant. Differences of < 1 Gy between all four techniques were categorized as “Any technique” being optimal. This was applied to each separate dose-related endpoint: MHD, MLD, and bilateral MBD. The overall optimal plan with regard to dose for each patient was then determined by tallying the endpoints: two for male and three for female patients. If results were equivocal, patients were assigned to the least recently implemented technique for a more conservative estimate.

## 3 Results

IMPT-BH resulted in the lowest average MHD at 5.4 Gy (*p* < 0.01) compared to VMAT-FB (8.0 Gy), VMAT-BH (6.3 Gy) and IMPT-FB (5.8 Gy). IMPT-BH also resulted in the lowest average MLD at 6.7 Gy (*p*<0.01), compared to VMAT-FB (9.2 Gy), VMAT-BH (7.5 Gy), and IMPT-FB (7.3 Gy). Average bilateral MBD was lower with IMPT (2.1 Gy) compared to VMAT (3.2 Gy; *p*<0.01). The BH technique itself did not lead to a significantly lower average MBD compared to the FB technique (Table 3, Table 4). The ranking of techniques from the lowest dose to the highest dose differed between individual patients (Supplementary Material, Figs. S2-S4).

**Table 3:** Average dose-related outcomes and NTCP values per technique.

***Metric, average***	***N***	**VMAT-FB**	**VMAT-BH**	**IMPT-FB**	**IMPT-BH**
		***Mean****(±SD)*	***Mean****(±SD)*	***Mean****(±SD)*	***Mean****(±SD)*
**MHD (Gy)**	58	**8.0** (4.1)	**6.3** (3.8) ‡	**5.8** (3.1) ‡	**5.4** (2.9) §
**MLD (Gy)**	58	**9.2** (3.2)	**7.5** (3.0) ‡	**7.3** (3.1) ‡	**6.7** (2.9) §
**MBD bilateral (Gy)**	29	**3.3** (2.1)	**3.1** (1.9)	**2.2** (1.8) ¥	**2.0** (1.4) ¥
**Integral dose (Gy/L)**	58	**143** (70)	**148** (80)	**120** (72) ¥	**123** (63) ¥
		***Median****(IQR)*	***Median****(IQR)*	***Median****(IQR)*	***Median****(IQR)*
**ACE (%)**	58	**19.9** (19)	**18.5** (18.6) ‡	**18.6** (14.9) ‡	**17.9** (15.7) §
**Sec Lung Cancer (%)**	13	**12.9** (4.6)	**10.3** (5.7) ‡	**8.9** (6.6) ‡	**8.0** (6.7) ¥
**Sec Breast Cancer (%)**	24	**5.2** (6.3)	**4.3** (7.0)	**2.7** (7.0) †	**2.3** (6.9) ¥

MHD = mean heart dose. MLD = mean lung dose. MBD bilateral = bilateral mean breast dose. VMAT = volumetric modulated arc therapy. IMPT = intensity modulated proton therapy. FB = free-breathing. BH = breath-hold. ACE = acute coronary events. Sec Lung Cancer = secondary lung cancer (smoking patients according to NIPP criteria, ≤50 yrs). Sec Breast Cancer = secondary breast cancer (female patients, ≤40 yrs), SD = standard deviation.

*Dose-related comparisons are performed with paired samples T-tests, and NTCP value comparisons with Wilcoxon signed-rank tests.*

§ Significantly different from VMAT-FB, VMAT-BH, and IMPT-FB (*p* < 0.01).

‡ Significantly different from VMAT-FB (*p* < 0.01).

¥ Significantly different from VMAT-FB and VMAT-BH (*p* < 0.01).

† Significantly different from VMAT-FB and VMAT-BH (*p* < 0.05).

**Table 4:** Mean difference in dose-related outcomes.

**MHD (Gy)**	**VMAT-FB**	**VMAT-BH**	**IMPT-FB**	**IMPT-BH**
**VMAT-FB**	**−**	**−1.7 (−2.2, −1.3) < 0.001**	**−2.2 (−2.8, −1.6) < 0.001**	**−2.7 (−3.2, −2.1) < 0.001**
**VMAT-BH**	**+1.7 (+1.3, +2.2) < 0.001**	**−**	−0.5 (−0.9, +0.0) 0.07	**−0.9 (−1.3, −0.5) < 0.001**
**IMPT-FB**	**+2.2 (+1.6, +2.8) < 0.001**	+0.5 (−0.0, +0.9) 0.07	**−**	**−0.5 (−0.7, −0.2) < 0.001**
**IMPT-BH**	**+2.7 (+2.1, +3.2) < 0.001**	**+0.9 (+0.5, +1.3) < 0.001**	**+0.5 (+0.2, +0.7) < 0.001**	**−**

**MLD (Gy)**	**VMAT-FB**	**VMAT-BH**	**IMPT-FB**	**IMPT-BH**

**VMAT-FB**	**−**	**−1.7 (−2.0, −1.5) < 0.001**	**−1.9 (−2.3, −1.6) < 0.001**	**−2.6 (−2.9, −2.2) < 0.001**
**VMAT-BH**	**+1.7 (+1.5, +2.0) < 0.001**	**−**	−0.2 (−0.5, +0.2) 0.30	**−0.8 (−1.1, −0.5) < 0.001**
**IMPT-FB**	**+1.9 (+1.6, +2.3) < 0.001**	+0.2 (−0.2, +0.5) 0.30	**−**	**−0.6 (−0.8, −0.4) < 0.001**
**IMPT-BH**	**+2.6 (+2.2, +2.9) < 0.001**	**+0.8 (+0.5, +1.1) < 0.001**	**+0.6 (+0.4, +0.8) < 0.001**	**−**

**MBD (Gy)**	**VMAT-FB**	**VMAT-BH**	**IMPT-FB**	**IMPT-BH**

**VMAT-FB**	**−**	**−**0.2 (−0.5, +0.1) 0.25	**−1.1 (−1.4, −0.8) < 0.001**	**−1.2 (−1.7, −0.8) < 0.001**
**VMAT-BH**	+0.2 (−0.1, +0.5) 0.25	**−**	**−0.9 (−1.2, −0.6) < 0.001**	**−1.1 (−1.4, −0.7) < 0.001**
**IMPT-FB**	**+1.1 (+0.8, +1.4) < 0.001**	**+0.9 (+0.6, +1.2) < 0.001**	**−**	−0.2 (−0.5, +0.2) 0.3
**IMPT-BH**	**+1.2 (+0.8. + 1.7) < 0.001**	**+1.1 (+0.7, +1.4) < 0.001**	+0.2 (−0.2, +0.5) 0.3	**−**

Data are shown in mean difference (95% CI); *p*-value.

Difference (in Gy) = reference (Y-axis) – comparator (X-axis).

*p* < 0.05 are shown in **bold**.

**Fig. 1:**
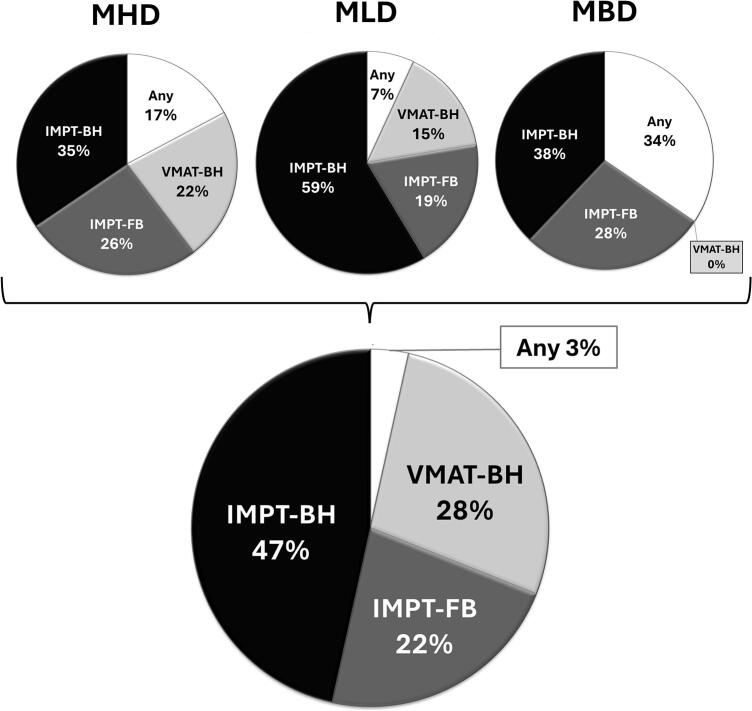
The optimal technique with regard to dose, per patient. Pie charts of the patient distribution per technique based on the mean heart dose (*n* = 58), mean lung dose (*n* = 58), and bilateral mean breast dose (*n* = 29; female patients). Each patient was assigned to the technique which was optimal with regard to dose for every separate dose-related endpoint, with an absolute dose difference of ≥ 1 Gy between any technique being considered clinically relevant. Differences of < 1 Gy between all four techniques were categorized as “Any technique” being optimal. The overall optimal plan with regard to dose for each patient was then determined by tallying the endpoints: two for male and three for female patients. If results were equivocal, patients were assigned to the least recently implemented technique for a more conservative estimate. MHD = mean heart dose. MLD = mean lung dose. MBD bilateral = bilateral mean breast dose. VMAT = volumetric modulated arc therapy. IMPT = intensity modulated proton therapy. FB = free-breathing. BH = breath-hold.

IMPT-BH reduced median NTCP for ACE to 17.9%, compared to VMAT-FB (19.9%), VMAT-BH (18.5%) and IMPT-FB (18.6%). IMPT-BH reduced median NTCP for SLC to 8.0%, compared to VMAT-FB (12.9%), VMAT-BH (10.3%) and IMPT-FB (8.9%). IMPT-BH (*p* < 0.01) and IMPT-FB (*p* < 0.05) resulted in lower median risks for SBC compared to VMAT (Table 4, Table 5). IMPT-BH was the optimal RT technique for MHD, MLD, and bilateral MBD in 35%, 59%, and 38% of patients, respectively (Fig. 1). Taken together with IMPT-FB, 61%, 78%, and 66% of patients benefited the most from any IMPT plan. Overall, IMPT-BH and IMPT-FB were the optimal techniques for ≥ 69% of patients (Fig. 1).

**Table 5:** Median difference in NTCP-related outcomes.

**ACE (%)**	**VMAT-FB**	**VMAT-BH**	**IMPT-FB**	**IMPT-BH**
**VMAT-FB**	**−**	**−1.3 (−2.1, −0.7) < 0.01**	**−1.3 (−1.8, −0.8) < 0.01**	**−1.7 (−2.8, −1.5) < 0.01**
**VMAT-BH**	**+1.3 (+0.7, +2.1) < 0.01**	**−**	0.0 (−0.6, +0.2) 0.12	**−0.3 (−0.7, −0.0) < 0.01**
**IMPT-FB**	**+1.3 (+0.8, +1.8) < 0.01**	0.0 (−0.2, +0.6) 0.12	**−**	**−0.2 (−0.6, −0.0) < 0.01**
**IMPT-BH**	**+1.7 (+1.5, +2.8) < 0.01**	**+0.3 (+0.0, +0.7) < 0.01**	**+0.2 (+0.0, +0.6) < 0.01**	**−**

**SLC (%)**	**VMAT-FB**	**VMAT-BH**	**IMPT-FB**	**IMPT-BH**

**VMAT-FB**	**−**	**−2.2 (−2.7, −1.2) < 0.01**	**−2.8 (−3.9, −2.1) < 0.01**	**−3.7 (−5.1, −2.2) < 0.01**
**VMAT-BH**	**+2.2 (+1.2, +2.7) < 0.01**	**−**	**−0.8 (−1.6, +0.1) 0.046**	**−1.6 (−2.3, −0.2) < 0.01**
**IMPT-FB**	**+2.8 (+2.1, +3.9) < 0.01**	**+0.8 (−0.1, +1.6) 0.046**	**−**	−0.3 (−0.8, +0.1) 0.10
**IMPT-BH**	**+3.7 (+2.2, +5.1) < 0.01**	**+1.6 (+0.2, +2.3) < 0.01**	+0.3 (−0.1, +0.8) 0.10	**−**

**SBC (%)**	**VMAT-FB**	**VMAT-BH**	**IMPT-FB**	**IMPT-BH**

**VMAT-FB**	**−**	−0.2 (−1.1, +0.3) 0.50	**−2.2 (−3.4, −1.3) < 0.01**	**−2.3 (−3.4, −1,5) < 0.01**
**VMAT-BH**	+0.2 (−0.3, +1.1) 0.50	**−**	**−1.8 (−2.9, −1.2) < 0.01**	**−1.6 (−3.6, −1,2) < 0.01**
**IMPT-FB**	**+2.2 (+1.3, +3.4) < 0.01**	**+1.8 (+1.2, +2.9) < 0.01**	**−**	−0.1 (−1.0, +0.4) 0.37
**IMPT-BH**	**+2.3 (+1.5, +3.4) < 0.01**	**+1.6 (+1.2, +3.6) < 0.01**	+0.1 (−0.4, +1.0) 0.37	**−**

Data are shown in median difference (95% CI, bootstrapped); *p*-value.

Difference (in %) = reference (Y-axis) – comparator (X-axis).

*p* < 0.05 are shown in **bold**.

## 4 Discussion

This in silico planning study compared four clinically available techniques for mediastinal lymphoma patients. IMPT-BH achieved the lowest average MHD, MLD, and bilateral MBD compared with VMAT-FB, VMAT-BH and IMPT-FB. Differences in MHD and MLD were statistically significant compared to all other techniques. Both IMPT techniques resulted in significantly lower MBD compared to VMAT. With regard to absolute dose, IMPT was optimal for the majority of patients.

These results aligned with our estimation that combining IMPT with BH would synergistically reduce the dose in OOIs, and support optimizing patient care by applying either one or both techniques [39]. Largest relative dose differences were seen in MBD compared to MHD and MLD due to three causes. Firstly, all IMPT-BH plans were optimized with a larger robustness margin (8 mm for IMPT-BH *vs* 5 mm for VMAT-BH) to account for potentially higher intrafraction instability due to longer treatment times. A margin analysis will evaluate the current margin and the feasibility of a margin reduction to further reduce the dose in OOIs. Secondly, the lymphoma is located closer to the lungs and heart. In the latter case it often encompasses a significant part of its circumference, while planning margins overlap with the heart. Although proton particles have a steeper dose fall-off, reducing MHD more effectively than photon therapy, MLD and MBD benefit relatively more, which aligns with the review by Patel et al [39]. Thirdly, even though the natures of VMAT and IMPT irradiation are different, the coverage criteria should be comparable. A calibration between the different target coverage and robustness criteria for the PTV and CTV voxel-wise minimum, respectively, was performed. The coverage was deemed comparable; similar to Korevaar et al [47]. In this study, IMPT coverage planning requirements were stricter, skewing DVH-metrics in favour of VMAT plans: the coverage in IMPT plans was robustly optimized on separate CTV subunits, i.e. neck (left and right), axilla (left and right), mediastinum (cranially and caudally located of the carina), and nodes directly located on the diaphragm, while the coverage in VMAT plans was optimized on the entire CTV. We adhered to current clinical requirements for this study. Future developments with less strict requirements on IMPT robustness optimization will further reduce OOI doses.

Another factor that relates to adequate coverage is the interplay effect between lung and soft tissues. This was mitigated by hyperfractionated treatment in the thorax and relatively large spot sizes of the Mevion Hyperscan S250i. Evaluating the effect of interplay in other thoracic sites without applying repainting, no relevant dose degradation was found [35], [50], [51], [52]. Risk of distal fall-off effects is mitigated by using multiple beam directions (smear-out effect) and two distal layers. Beam angles were chosen in such way that they are positioned tangential to enable organ avoidance.

This study provides evidence that IMPT-BH yields the lowest average dose in the most relevant OOIs, compared to the other three most prevalent techniques. Several in silico studies reported IMPT-BH achieving dose reduction compared to one or two other techniques [37], [40], [41], [42], [43], [44], [45], [53]. However, IMPT-BH was not clinically implemented at time of these publications, and it was not clarified whether plans met clinical requirements. Plan comparison data from RT centers that did clinically implement IMPT-BH are scarce. These centers reported on adequate plan robustness and breath-hold duration [54]; average intra-fraction reproducibility (<1 mm and < 1 degree); minimal DVH-metric differences in CTV coverage for inter-fraction setup variation and anatomical changes [55]; the effect of IMPT-BH on glandular breast dose [56]; acute side effects and survival outcomes [57]. These are in line with preliminary results from unpublished data [58], [59].

Currently, the model-based selection method is used to qualify patients for proton therapy in the Netherlands. The pros and cons of using NTCP models are covered in other publications [49], [60]. To estimate the potential long-term clinical impact of reducing OOI doses, NTCP values for ACE, SLC, and SBC were calculated. Since DVH-metrics are used in the models, IMPT-BH significantly reduced the average estimated risk of all three late side effects. Given the young age of ML patients, minimizing late effects by selecting the technique with the lowest expected risk for the individual patient is crucial [61]. The optimal plan for each individual patient depends on patient, disease and treatment characteristics. It is hypothesized that sex and target volume, comprised of certain (combinations of) involved anatomic regions, play an important role in DVH-metric outcomes. The treatment planning process is time-consuming, requiring at least two RT plans for model-based selection. Therefore, a decision support tool is needed to optimize plan selection and better predict which technique provides the lowest doses to the OOIs for each patient. A larger patient cohort is needed to identify predictive characteristics that are associated with a certain technique, which is subject of ongoing research.

Several potential sources of bias should be considered when interpreting these results. Patient and technique selection biases, because a small minority of ML patients did not undergo BH-CT simulation. This was due to the clinician’s estimation that thoracic OOIs would receive low doses and/or that the BH benefit would be too slight, e.g. nodal involvement cranial of the heart. Despite this estimation, treating physicians tended to make a BH plan to quantify the dose differences compared to FB, just in case. Furthermore, inter-planner variability bias might not have yielded the optimal plan for the study, even though all RTTs were experienced in making plans for lymphoma patients. This reflects clinical variation between RTTs and RT centers, and was deemed acceptable for this study. Ongoing peer sessions to compare planning techniques between RTTs could further improve RT plans. Additionally, to mitigate prioritization bias in this study, RTTs used institute guidelines on prioritizing and balancing the sparing of multiple OOIs, while maintaining coverage, and minimizing volumes with doses > 107% in the voxel-wise max. All plans met requirements for clinical use. Also, differential evaluation bias could have been introduced, since the clinically used IMPT-FB plans were robustly optimized on the average CT and additionally evaluated on the 4D-CT, while the in silico IMPT-FB plans were not evaluated in 4D-CT. In clinical practice, 4D evaluations rarely led to IMPT plans failing robustness criteria or exceeding dose guidance [62]. RTTs would recompute the plans to meet these criteria. Since this was not applied to the in silico plans, it could have led to more favourable doses to the OOIs in IMPT-FB plans for this analysis. Another source of bias is based on the method of classification: to analyze individual differences, each patient was assigned to one of four techniques per separate dose-related endpoint, with an absolute dose difference of ≥ 1 Gy between any technique considered clinically relevant [39]. This threshold aligns with publications citing dose reductions of ≥ 1 Gy as clinically meaningful [29], [30], [32], [34], [35], [36], [37], [38], [39] and daily clinical practice. Finally, the aforementioned different margins introduced a technique selection bias.

In conclusion, this plan comparison study using clinically acceptable treatment plans demonstrates that IMPT-BH yields the most favorable overall OOI sparing for mediastinal lymphoma patients. These dose-related advantages have the potential to best reduce the risk of treatment-related late effects in this young patient population. While IMPT-BH and IMPT-FB were the optimal techniques for at least 69% of patients, the optimal technique varied on an individual basis. This underscores plan technique selection by identifying predictive patient, disease and anatomical characteristics. Further technical developments and clinical experience with IMPT-BH may enhance dose-related benefits, and contribute to long-term health outcomes for lymphoma patients.

## Declaration of generative AI and AI-assisted technologies in the writing process

Statement: During the preparation of this work the author(s) used Microsoft Copilot in order to improve readability of the text. After using this tool/service, the author(s) reviewed and edited the content as needed and take(s) full responsibility for the content of the published article.

## CRediT authorship contribution statement

**Bastiaan D.P. Ta:** Writing – review & editing, Writing – original draft, Visualization, Validation, Supervision, Project administration, Methodology, Investigation, Formal analysis, Data curation, Conceptualization. **Richard A.M. Canters:** Writing – review & editing, Writing – original draft, Visualization, Supervision, Software, Resources, Methodology, Investigation, Formal analysis, Data curation, Conceptualization. **Kim van der Klugt:** Writing – review & editing, Project administration, Investigation, Data curation, Conceptualization. **Gloria Vilches-Freixas:** Writing – review & editing, Writing – original draft, Visualization, Software, Resources, Methodology, Investigation, Data curation, Conceptualization. **Esther Kneepkens:** Writing – review & editing, Investigation, Conceptualization. **Fleur Vereijken:** Writing – review & editing, Data curation. **Maud Cobben:** Writing – review & editing, Data curation, Conceptualization. **Maud van den Bosch:** Writing – review & editing, Data curation. **Indra Lubken:** Writing – review & editing, Data curation. **Cissy Stultiens:** Writing – review & editing, Data curation. **Marije Velders:** Writing – review & editing, Data curation. **Tina Verstappen:** Writing – review & editing, Data curation. **Gyanne Tholen:** Writing – review & editing, Data curation. **Anne G.H. Niezink:** Writing – review & editing, Writing – original draft, Visualization, Methodology. **Dirk K.M. De Ruysscher:** Writing – review & editing, Writing – original draft, Visualization, Supervision, Methodology, Conceptualization. **Maaike Berbée:** Writing – review & editing, Writing – original draft, Visualization, Supervision, Methodology, Conceptualization.

## Declaration of competing interest

The authors declare that they have no known competing financial interests or personal relationships that could have appeared to influence the work reported in this paper.

## Appendix A Supplementary data

Supplementary Data 1 Treatment preparation details and individual patient dose outcomes.
