# Supplementary material for: Improved organs-of-interest dose reduction potential with intensity modulated proton therapy and breath-hold in mediastinal lymphoma
Source: Phys Imaging Radiat Oncol. 2026 May 10;39:100996. doi: 10.1016/j.phro.2026.100996 (PMC13196566; doi:10.1016/j.phro.2026.100996)
Supplement: Supplementary Data 1 — Treatment preparation details and individual patient dose outcomes. [file mmc1.pdf]

Supplementary Material

Improved organs-of-interest dose reduction potential with intensity modulated proton therapy and breath-hold in mediastinal lymphoma

A. Immobilization devices and planning CT simulation

All patients underwent Computer Tomography (CT) simulation in supine position with the Siemens SOMATOM Drive or Confidence CT scanner (Siemens Healthineers, Forchheim, Germany). Before clinical implementation of IMPT-BH, patients were either immobilized with a 5-points head, neck and shoulders mask (Orfit, Belgium) if neck or periclavicular nodes were involved, or otherwise positioned in a Monarch Overhead Arm Positioner (CQ Medical, US). After IMPT-BH implementation, all patients were positioned on an Evolve wedge board (Cablon Medical, The Netherlands) with a 7.5 degree incline to improve the field of view of the surface-scanning system. In case of neck or periclavicular nodal involvement, a chin and thorax strap were used for stabilization.

CT acquisition for RT planning was performed before and after chemotherapy, enabling involved site RT. CT scans included a respiratory-controlled four-dimensional CT scan (4D-CT) with respiratory gating system (Anzai Medical, Japan), a planning CT in BH, and three consecutive BH CTs to visually assess BH stability. Slice thickness for all scans was three millimeters. Iodinated contrast was used by clinician's preference for delineation purposes, but not on CT scans used for proton planning; to avoid density changes.

B. Breath hold technique and image guidance with surface-scanning

A voluntary moderately deep BH technique was used to mitigate motion. Patients received verbal instructions on the BH technique prior to CT acquisition by an RTT experienced with treating patients with this technique. Patients received goggles for live visual feedback of their own breathing window in BH, which was 3 mm. During BH scan acquisition three additional BH scans (BH #1-2-3) were made.

Patients were able to adjust the BH voluntarily. Breath-holds were monitored with a surface scanning system (C-RAD Sentinel, Sweden). The Airvo nasal high-flow therapy system (Fisher&Paykel, New Zealand) provided humidified air flow of 40 liters/min with 21% oxygen concentration.

C. Radiotherapy delineation and planning

Target volume delineation was done by radiation oncologists (RO) and a physician assistant (PA), experienced in the field of hematological malignancies. Contour delineation was performed on the BH scan and was checked by an experienced colleague.

The pre-chemotherapy gross tumor volume (GTV) was delineated on the pre-chemotherapy BH-CT scans, copied to the post-chemotherapy BH-CT scan, and adjusted to define the clinical target volume (CTV) according to ILROG guidelines, i.e. involved site [1–4]. In primary RT patients involved field RT was used. A boost CTV was created for a simultaneous integrated boost in consolidation or salvage setting, where applicable.

For the FB plans, to account for motion, the CTV was propagated (with deformable image registration) from the BH-CT to the 50ex phase of the 4D-CT. The delineation was then manually adjusted and propagated again (with deformable image registration) to the other seven respiratory phases on the 4D-CT. The CTVs were then manually adjusted.

For IMPT plans, the entire CTV was then divided into subunits for 4D evaluation, i.e. neck (left/right), axilla (left/right), mediastinum (cranial/caudal to the carina), and nodes directly located on the diaphragm.
For VMAT-FB and VMAT-BH in the thoracic region, the CTV was expanded using an 8 or 5 mm isotropic margin, respectively, to create a PTV. The radiation oncologist visually checked whether the target volume on the BH #1-2-3 scans still fell within the PTV margin on the primary BH scan. In the neck, periclavicular and axillary regions, the isotropic PTV margin was 5 mm. A 7 mm PTV margin in the thorax was adequate for VMAT-FB and 5 mm for VMAT-BH, based on Canters et al, Table S2 [5]. In clinical practice, it was decided an ITV was not necessary.
For IMPT-FB and IMPT-BH, the robustness margin was 8 mm in the thoracic region, and 5 mm in the neck, periclavicular and axillary region. A larger robustness margin of 8 mm in the thorax was for the IMPT-BH (compared to VMAT-BH, 5 mm) to account for potentially higher intrafraction instability due to longer treatment times, until a margin analysis has been performed.
Relevant organs-of-interest (OOIs), e.g. parotid glands, submandibular glands, thyroid gland, spinal canal, humeral heads, lungs, heart and breasts, were delineated by the RTT and checked by the RO or PA.

D. Radiotherapy planning

Coverage criteria and clinical goals are shown below in Tables S1 and S2, respectively [4]. For IMPT plans, a dose grid resolution of 0.3 cm^3, and dose calculation algorithm Monte Carlo (v5.4) in the RayStation TPS (RaySearch Laboratories v.12A, Sweden) were used.
Before IMPT was introduced for ML patients, the relationship between PTV nominal coverage and CTV voxel-wise minimum coverage was calibrated, according to Korevaar et al [6]. Twenty ML photon plans based on the PTV were robustly evaluated in the TPS (Eclipse, Varian). The PTV D99 was plotted against the CTV voxel-wise minimum D98 (Supplementary Figure S1). From this plot, the criterion “PTV $D_{98} \geq 95\%$ of the prescription dose” corresponded to “CTV voxel-wise minimum $D_{98} \geq 94\%$ of the prescription dose.” Therefore, in this planning study, the coverage criteria between the PTV-based photon plans and the CTV-robust based proton plans were deemed similar.
Most clinical goals are not defined as definitive constraints, as the biological doses for lymphoma patients are well below the organ tolerances. To further optimize the plans, in clinical practice we already made use of criteria for the RTT to strive towards. We used an initial prioritization, stipulated in our centre’s guidelines (heart – breasts – lungs – thyroid – esophagus – spinal canal – humeral head) to guide RTTs in optimizing between these organs. The prioritization could be changed, e.g. favouring breasts over heart, in individual cases after consulting with the clinician. Additional OARs: based on the anatomic region involved we also generated structures for brain stem, mandibula, oral cavity, parotid glands, submandibular glands.
The additional plans made by the experienced RTTs were not formally approved by the clinician, but the RTTs applied the same treatment planning process as for clinically used plans. Deliberation with the clinician and/or physicist was possible with challenging plans.

Table S1. Coverage criteria

**VMAT plans**	• V_{95} PTV≥99% (PTV1 and, where applicable, PTV2).<br>• Dose guidance ≤107%.
**IMPT plans**	• V_{94} CTV≥98% in voxel-wise minimum plan.<br>• Dose guidance ≤107% in nominal plan.<br>• Dose guidance ≤110% in voxel-wise maximum plan.

Table S2. Clinical goals

**Organ-at-risk**	**DVH-metric**	**Strive towards / *Dose guidance***
Heart	D_{mean}	<10 Gy / *<15 Gy*<br>In general, as low as possible
Aortic valve	D_{mean}	<20 Gy
Mitral valve	D_{mean}	<20 Gy
Breast, left or right	D_{mean}<br>$V_{4\,Gy}$ (cm^3)	<4 Gy<br>As low as possible
Lungs	D_{mean}<br>$V_{5\,Gy}$<br>$V_{20\,Gy}$<br>$V_{30\,Gy}$	<13.5 Gy<br><55%<br><30%<br>As low as possible
Esophagus	D_{mean}<br>$V_{30\,Gy}$	As low as possible<br>As low as possible
Spinal cord	D_{max}<br>D_{max}	<105%<br>*<54 Gy*
Thyroid	D_{mean}<br>D_{max}	<20 Gy<br>As low as possible
Humeral head	$V_{20\,Gy}$<br>$V_{15\,Gy}$	<20%<br>*<40%*
Submandibular gland, left or right<br><br>Submandibular glands	D_{mean}<br>D_{mean}<br>D_{mean}	Ipsilateral <5 Gy<br>Contralateral <2 Gy<br>*<30 Gy*
Parotid gland, left or right	D_{mean}<br>D_{mean}<br>D_{mean}	Ipsilateral <5 Gy<br>Contralateral <2 Gy<br>*<25 Gy*
Oral cavity	D_{mean}	As low as possible
Brain stem	D_{max}<br>D_{max}	<105%<br>*<54 Gy*

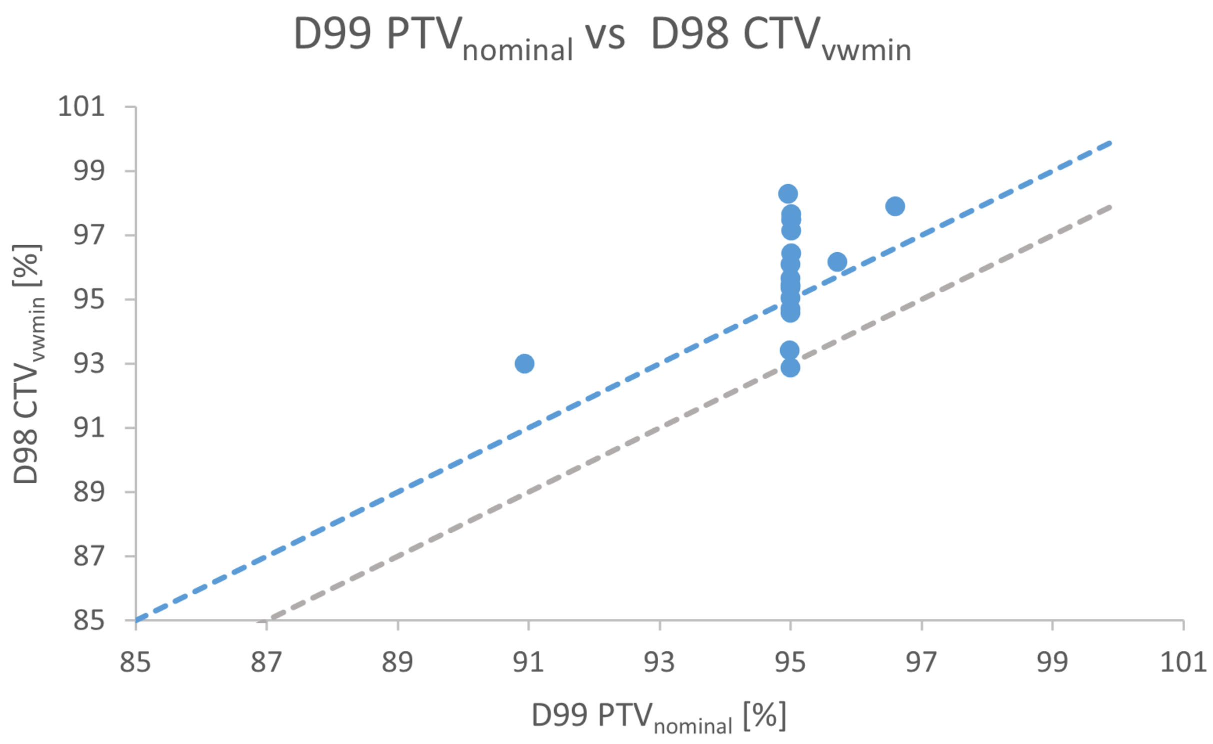

Figure S1. Calibration between VMAT and IMPT coverage criteria: PTV D99 was plotted against the CTV voxel-wise minimum (CTV_{vwmin}) D98

E. Normal tissue complication probability calculation

The excess relative risk (ERR) of ACE was calculated with the model of Darby et al with a linear dose-effect relationship of 7.4%/Gy MHD in breast cancer patients, based on the individual patient's MHD, age, sex and cardiovascular risk factors [7,8]. The risk of secondary lung cancer for patients ≤50 years of age at time of RT, who smoked, ceased smoking <1 year prior to RT, or had smoked ≥20 pack-years, was calculated with an ERR of 11%/Gy MLD reported by Taylor et al [9]. The risk for secondary breast cancer for female patients ≤40 years of age at time of RT was calculated based on the dose-effect relationship reported by Roberti et al with an ERR of 19%/Gy MBD in Hodgkin lymphoma patients, and by Hooning et al. with an ERR of 21%/Gy MBD (95% CI 1-61%) in breast cancer patients [10,11].

Figures S2-4. Absolute dose reduction per patient compared to the VMAT-FB technique

Waterfalls plots showing the reduction of mean heart dose (Supplementary Figure S2, *n*=58), mean lung dose (Supplementary Figure S3, *n*=58) and bilateral mean breast dose (Supplementary Figure S4, *n*=29) for each patient. Every separate technique (VMAT-BH, IMPT-FB and IMPT-BH) was compared to the VMAT-FB dose (shown below in Gy). The patients were sorted in order of largest reduction of dose with IMPT-BH compared to VMAT-FB. Thus, patient numbering *between* these three supplementary figures to not correspond to the same patient.

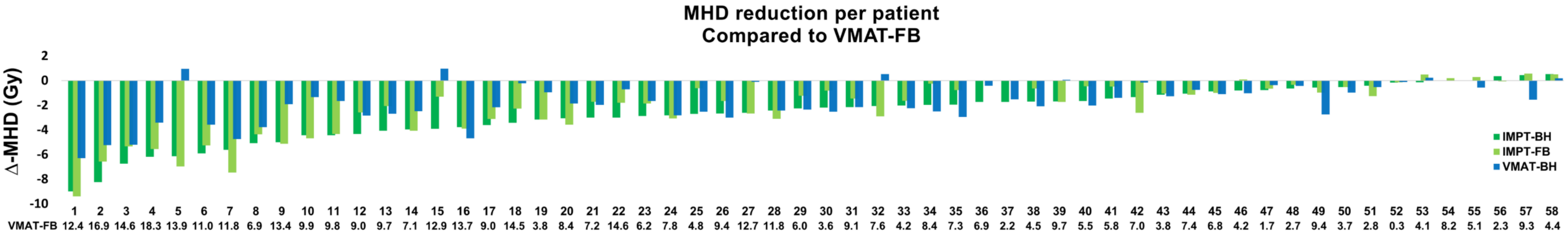

Figure S2

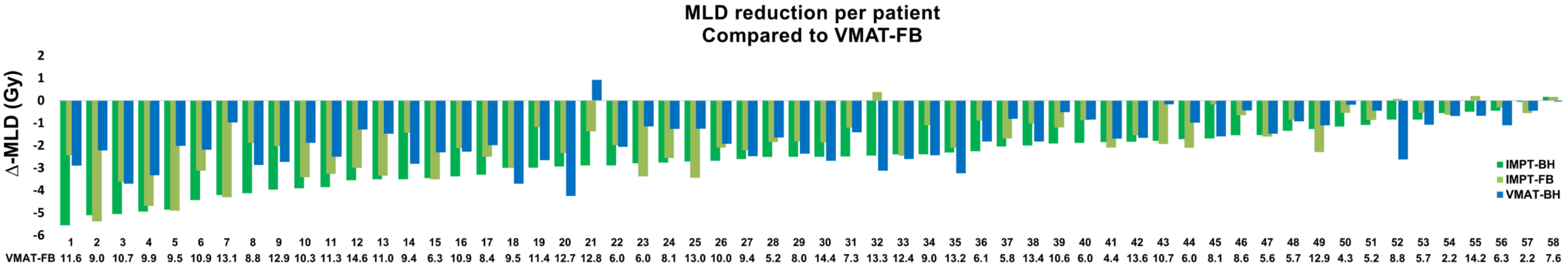

Figure S3

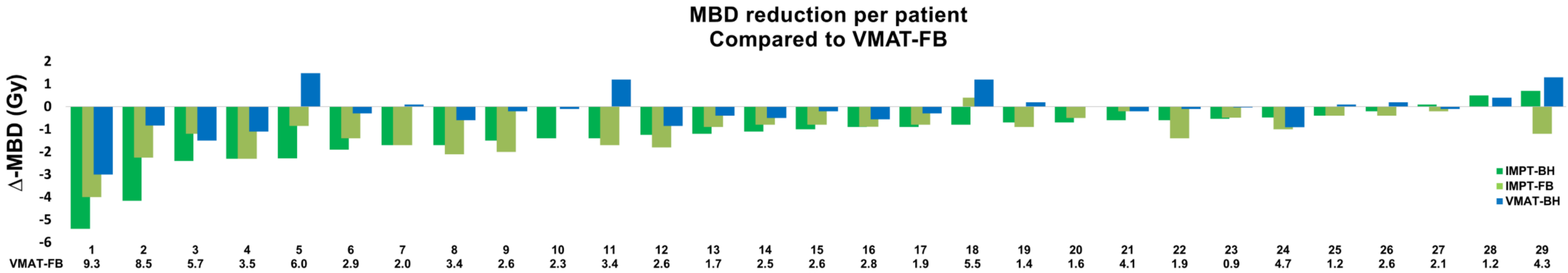
MBD reduction per patient
Compared to VMAT-FB
Δ-MBD (Gy)
2
1
0
-1
-2
-3
-4
-5
-6
IMPT-BH
IMPT-FB
VMAT-BH
1 2 3 4 5 6 7 8 9 10 11 12 13 14 15 16 17 18 19 20 21 22 23 24 25 26 27 28 29
VMAT-FB 9.3 8.5 5.7 3.5 6.0 2.9 2.0 3.4 2.6 2.3 3.4 2.6 1.7 2.5 2.6 2.8 1.9 5.5 1.4 1.6 4.1 1.9 0.9 4.7 1.2 2.6 2.1 1.2 4.3

Figure S4

References

[1] Yahalom J, Illidge T, Specht L, Hoppe RT, Li YX, Tsang R, et al. Modern radiation therapy for extranodal lymphomas: Field and dose guidelines from the international lymphoma radiation oncology group. Int J Radiat Oncol Biol Phys 2015;92:11–31. https://doi.org/10.1016/j.ijrobp.2015.01.009.

[2] Illidge T, Specht L, Yahalom J, Aleman B, Berthelsen AK, Constine L, et al. Modern radiation therapy for nodal non-hodgkin lymphoma - Target definition and dose guidelines from the international lymphoma radiation oncology group. Int J Radiat Oncol Biol Phys 2014;89:49–58. https://doi.org/10.1016/j.ijrobp.2014.01.006.

[3] Specht L, Yahalom J, Illidge T, Berthelsen AK, Constine LS, Eich HT, et al. Modern radiation therapy for Hodgkin lymphoma: Field and dose guidelines from the international lymphoma radiation oncology group (ILROG). Int J Radiat Oncol Biol Phys 2014;89:854–62. https://doi.org/10.1016/j.ijrobp.2013.05.005.

[4] Wirth A, Mikhaeel NG, Aleman BMP, Pinnix CC, Constine LS, Ricardi U, et al. Involved Site Radiation Therapy in Adult Lymphomas: An Overview of International Lymphoma Radiation Oncology Group Guidelines. Int J Radiat Oncol Biol Phys 2020;107:909–33. https://doi.org/10.1016/j.ijrobp.2020.03.019.

[5] Canters R, Vaassen F, Lubken I, Cobben M, Murrer L, Peeters S, et al. Radiotherapy for mediastinal lymphoma in breath hold using surface monitoring and nasal high flow oxygen: Clinical experiences and breath hold stability. Radiother Oncol 2023;183:109594. https://doi.org/10.1016/j.radonc.2023.109594.

[6] Korevaar EW, Habraken SJM, Scandurra D, Kierkels RGJ, Unipan M, Eenink MGC, et al. Practical robustness evaluation in radiotherapy – A photon and proton-proof alternative to PTV-based plan evaluation. Radiother Oncol 2019;141:267–74. https://doi.org/10.1016/j.radonc.2019.08.005.

[7] Darby SC, Ewertz M, McGale P, Bennet AM, Blom-Goldman U, Brønnum D, et al. Risk of Ischemic Heart Disease in Women after Radiotherapy for Breast Cancer. N Engl J Med 2013;368:987–98. https://doi.org/10.1056/nejmoa1209825.

[8] Van den Bogaard VAB, Ta BDP, Van der Schaaf A, Bouma AB, Middag AMH, Bantema-Joppe EJ, et al. Validation and modification of a prediction model for acute cardiac events in patients with breast cancer treated with radiotherapy based on three-dimensional dose distributions to cardiac substructures. J Clin Oncol 2017;35:1171–8. https://doi.org/10.1200/JCO.2016.69.8480.

[9] Taylor C, Duane FK, Dodwell D, Gray R, Wang Z, Wang Y, et al. Estimating the Risks of Breast cancer radiotherapy: Evidence from modern radiation doses to the lungs and Heart and From previous randomized trials. J Clin Oncol 2017;35:1641–9. https://doi.org/10.1200/JCO.2016.72.0722.

[10] Roberti S, Van Leeuwen FE, Ronckers CM, Krul IM, De Vathaire F, Veres C, et al. Radiotherapy-Related Dose and Irradiated Volume Effects on Breast Cancer Risk among Hodgkin Lymphoma Survivors. J Natl Cancer Inst 2022;114:1270–8. https://doi.org/10.1093/jnci/djac125.

[11] Hooning MJ, Aleman BMP, Hauptmann M, Baaijens MHA, Klijn JGM, Noyon R, et al. Roles of radiotherapy and chemotherapy in the development of contralateral breast cancer. J Clin Oncol 2008;26:5561–8. https://doi.org/10.1200/JCO.2007.16.0192.
